# Supplementary material for: Hot spot prediction in protein-protein interactions by an ensemble system
Source: BMC Syst Biol. 2018 Dec 31;12(Suppl 9):132. doi: 10.1186/s12918-018-0665-8 (PMC6311905; doi:10.1186/s12918-018-0665-8)
Supplement: Supplementary file 2 — All relevant MATLAB code. A simple Matlab implement of our predictor. (ZIP 19,742 kb) [file 12918_2018_665_MOESM2_ESM.zip › Additional file 2/Read ASA/ASA website/dbMPIKT/4P5TC.html]

 NetSurfP - Protein Surface Accessibility and Secondary Structure Predictions

  

|  |  |  |
| --- | --- | --- |
|  |  | NetSurfP - Protein Surface Accessibility and Secondary Structure Predictions  Technical University of Denmark |

  


---

```
# For publication of results, please cite:
# A generic method for assignment of reliability scores applied to solvent accessibility predictions.
# Bent Petersen, Thomas Nordahl Petersen, Pernille Andersen, Morten Nielsen and Claus Lundegaard
# BMC Structural Biology 2009, 9:51 doi:10.1186/1472-6807-9-51
#
# Column 1: Class assignment - B for buried or E for Exposed - Threshold: 25% exposure, but not based on RSA
# Column 2: Amino acid
# Column 3: Sequence name
# Column 4: Amino acid number
# Column 5: Relative Surface Accessibility - RSA
# Column 6: Absolute Surface Accessibility
# Column 7: Z-fit score for RSA prediction
# Column 8: Probability for Alpha-Helix
# Column 9: Probability for Beta-strand
# Column 10: Probability for Coil
E I  4P5TC                  1    0.611 112.961  -0.836   0.003   0.003   0.994
E E  4P5TC                  2    0.595 103.929   0.543   0.004   0.197   0.799
E A  4P5TC                  3    0.353  38.934  -0.006   0.022   0.359   0.619
E D  4P5TC                  4    0.589  84.860   0.122   0.022   0.359   0.619
B H  4P5TC                  5    0.214  38.999   0.415   0.022   0.552   0.426
B V  4P5TC                  6    0.226  34.782   0.345   0.001   0.900   0.099
B G  4P5TC                  7    0.131  10.302   0.669   0.001   0.959   0.040
B T  4P5TC                  8    0.099  13.801   0.676   0.001   0.959   0.040
B Y  4P5TC                  9    0.062  13.314   0.731   0.001   0.900   0.099
B G  4P5TC                 10    0.038   2.975  -0.634   0.002   0.816   0.182
B I  4P5TC                 11    0.026   4.736   0.897   0.001   0.900   0.099
B S  4P5TC                 12    0.063   7.419  -0.332   0.001   0.959   0.040
B V  4P5TC                 13    0.042   6.409   0.785   0.001   0.959   0.040
B Y  4P5TC                 14    0.121  25.815   0.301   0.002   0.816   0.182
E Q  4P5TC                 15    0.304  54.348  -0.590   0.004   0.514   0.481
E S  4P5TC                 16    0.532  62.386  -2.047   0.005   0.045   0.951
E P  4P5TC                 17    0.600  85.154  -2.839   0.003   0.003   0.994
E G  4P5TC                 18    0.403  31.755  -1.044   0.005   0.045   0.951
B D  4P5TC                 19    0.270  38.864  -1.553   0.004   0.085   0.910
E I  4P5TC                 20    0.474  87.635  -0.167   0.005   0.262   0.733
B G  4P5TC                 21    0.139  10.971  -0.254   0.003   0.718   0.279
B Q  4P5TC                 22    0.099  17.610   0.276   0.001   0.959   0.040
B Y  4P5TC                 23    0.161  34.427   0.139   0.001   0.959   0.040
B T  4P5TC                 24    0.136  18.863   1.330   0.000   0.983   0.017
B F  4P5TC                 25    0.065  13.025   0.738   0.001   0.959   0.040
B E  4P5TC                 26    0.067  11.670  -0.264   0.001   0.959   0.040
B F  4P5TC                 27    0.080  15.996   0.537   0.001   0.900   0.099
E D  4P5TC                 28    0.306  44.152   0.579   0.005   0.262   0.733
E G  4P5TC                 29    0.338  26.561  -0.819   0.018   0.047   0.935
E D  4P5TC                 30    0.449  64.629   0.224   0.022   0.359   0.619
B E  4P5TC                 31    0.173  30.293  -0.013   0.021   0.756   0.223
B L  4P5TC                 32    0.101  18.456   0.783   0.018   0.846   0.136
B F  4P5TC                 33    0.066  13.146   0.754   0.011   0.918   0.071
B Y  4P5TC                 34    0.117  24.939   1.142   0.011   0.918   0.071
B V  4P5TC                 35    0.035   5.395   0.596   0.011   0.918   0.071
B D  4P5TC                 36    0.167  24.065  -0.428   0.021   0.756   0.223
B L  4P5TC                 37    0.159  29.113   0.021   0.022   0.359   0.619
E D  4P5TC                 38    0.610  87.887   0.929   0.019   0.141   0.840
E K  4P5TC                 39    0.563 115.850   0.365   0.018   0.047   0.935
E K  4P5TC                 40    0.531 109.165  -0.901   0.018   0.047   0.935
E E  4P5TC                 41    0.418  73.007  -0.071   0.022   0.359   0.619
B T  4P5TC                 42    0.194  26.866   0.123   0.021   0.756   0.223
B V  4P5TC                 43    0.215  33.015  -0.027   0.021   0.756   0.223
B W  4P5TC                 44    0.140  33.622   0.143   0.021   0.756   0.223
E M  4P5TC                 45    0.284  56.788  -0.166   0.022   0.552   0.426
B L  4P5TC                 46    0.154  28.216  -0.475   0.021   0.279   0.699
E P  4P5TC                 47    0.380  53.950  -0.765   0.191   0.086   0.723
E E  4P5TC                 48    0.405  70.701  -0.797   0.354   0.048   0.598
E F  4P5TC                 49    0.318  63.762  -0.516   0.622   0.015   0.363
B G  4P5TC                 50    0.186  14.630  -1.718   0.622   0.015   0.363
E Q  4P5TC                 51    0.462  82.531  -0.133   0.622   0.015   0.363
B L  4P5TC                 52    0.283  51.799   0.160   0.717   0.014   0.269
B A  4P5TC                 53    0.171  18.844  -0.179   0.717   0.014   0.269
E S  4P5TC                 54    0.363  42.567  -0.642   0.622   0.015   0.363
E F  4P5TC                 55    0.330  66.291  -0.244   0.622   0.015   0.363
E D  4P5TC                 56    0.363  52.251   0.362   0.660   0.049   0.291
B P  4P5TC                 57    0.283  40.186  -0.154   0.717   0.014   0.269
E Q  4P5TC                 58    0.357  63.814  -0.796   0.717   0.014   0.269
E G  4P5TC                 59    0.474  37.320  -1.255   0.622   0.015   0.363
E G  4P5TC                 60    0.450  35.384  -0.979   0.522   0.016   0.462
E L  4P5TC                 61    0.368  67.454  -0.804   0.354   0.048   0.598
E Q  4P5TC                 62    0.454  81.067  -0.348   0.278   0.093   0.628
B N  4P5TC                 63    0.225  32.969  -0.022   0.125   0.227   0.648
B I  4P5TC                 64    0.278  51.411   0.318   0.069   0.386   0.545
B A  4P5TC                 65    0.135  14.888  -0.819   0.074   0.484   0.442
B V  4P5TC                 66    0.164  25.284  -0.097   0.087   0.683   0.230
B V  4P5TC                 67    0.034   5.195   0.246   0.087   0.683   0.230
E K  4P5TC                 68    0.309  63.541   0.412   0.079   0.592   0.329
B H  4P5TC                 69    0.314  57.117  -1.334   0.148   0.418   0.435
B N  4P5TC                 70    0.294  43.071  -0.855   0.125   0.227   0.648
E L  4P5TC                 71    0.396  72.544  -1.049   0.118   0.150   0.732
E G  4P5TC                 72    0.404  31.779  -0.751   0.066   0.296   0.638
E V  4P5TC                 73    0.370  56.869  -0.426   0.069   0.386   0.545
B L  4P5TC                 74    0.210  38.433  -0.265   0.069   0.386   0.545
B T  4P5TC                 75    0.227  31.513  -0.612   0.069   0.386   0.545
E K  4P5TC                 76    0.324  66.647  -1.445   0.069   0.386   0.545
E R  4P5TC                 77    0.380  87.066  -1.187   0.066   0.296   0.638
B S  4P5TC                 78    0.261  30.636  -1.336   0.066   0.296   0.638
E N  4P5TC                 79    0.455  66.612  -0.875   0.066   0.296   0.638
E S  4P5TC                 80    0.465  54.510  -0.989   0.064   0.216   0.721
E T  4P5TC                 81    0.365  50.695  -1.135   0.066   0.296   0.638
E P  4P5TC                 82    0.381  54.135  -1.423   0.064   0.216   0.721
E A  4P5TC                 83    0.419  46.174  -1.383   0.021   0.279   0.699
E T  4P5TC                 84    0.381  52.914  -1.140   0.022   0.359   0.619
E N  4P5TC                 85    0.392  57.389  -1.203   0.022   0.552   0.426
E E  4P5TC                 86    0.402  70.194  -1.646   0.004   0.420   0.576
E A  4P5TC                 87    0.340  37.512   0.187   0.005   0.262   0.733
B P  4P5TC                 88    0.119  16.914  -1.144   0.005   0.336   0.660
E Q  4P5TC                 89    0.258  46.007   0.042   0.002   0.816   0.182
B A  4P5TC                 90    0.017   1.851   0.838   0.001   0.900   0.099
B T  4P5TC                 91    0.157  21.845   0.528   0.001   0.959   0.040
B V  4P5TC                 92    0.024   3.643   0.001   0.001   0.900   0.099
B F  4P5TC                 93    0.100  20.110   0.127   0.018   0.846   0.136
B P  4P5TC                 94    0.145  20.547  -1.150   0.022   0.552   0.426
B K  4P5TC                 95    0.265  54.613  -0.193   0.020   0.205   0.775
E S  4P5TC                 96    0.384  44.970  -1.343   0.019   0.141   0.840
E P  4P5TC                 97    0.401  56.916  -1.533   0.019   0.141   0.840
E V  4P5TC                 98    0.444  68.212  -2.202   0.019   0.141   0.840
E L  4P5TC                 99    0.410  75.071  -1.437   0.018   0.088   0.893
E L  4P5TC                100    0.347  63.609  -2.027   0.018   0.088   0.893
E G  4P5TC                101    0.444  34.911  -1.586   0.052   0.084   0.864
E Q  4P5TC                102    0.437  78.066  -1.124   0.052   0.084   0.864
E P  4P5TC                103    0.391  55.540  -0.394   0.125   0.227   0.648
B N  4P5TC                104    0.111  16.250  -1.203   0.159   0.516   0.325
B T  4P5TC                105    0.173  24.037   0.535   0.273   0.587   0.140
B L  4P5TC                106    0.045   8.294  -0.011   0.273   0.587   0.140
B I  4P5TC                107    0.127  23.476   0.060   0.265   0.667   0.069
B C  4P5TC                108    0.098  13.717  -0.176   0.265   0.667   0.069
B F  4P5TC                109    0.136  27.235   0.523   0.265   0.667   0.069
B V  4P5TC                110    0.030   4.657  -0.779   0.268   0.505   0.227
B D  4P5TC                111    0.261  37.639  -0.414   0.135   0.317   0.548
E N  4P5TC                112    0.471  68.910  -0.624   0.019   0.141   0.840
B I  4P5TC                113    0.131  24.198  -1.148   0.019   0.141   0.840
E F  4P5TC                114    0.280  56.256  -0.034   0.005   0.045   0.951
B P  4P5TC                115    0.162  23.030  -0.707   0.005   0.045   0.951
E P  4P5TC                116    0.457  64.891  -0.610   0.004   0.138   0.858
E V  4P5TC                117    0.384  58.990  -0.298   0.004   0.514   0.481
B I  4P5TC                118    0.029   5.346   0.222   0.001   0.900   0.099
B N  4P5TC                119    0.252  36.966   0.826   0.001   0.959   0.040
B I  4P5TC                120    0.027   5.013   0.821   0.000   0.983   0.017
B T  4P5TC                121    0.184  25.549   1.777   0.000   0.983   0.017
B W  4P5TC                122    0.058  13.925   0.802   0.001   0.959   0.040
E L  4P5TC                123    0.254  46.489   1.074   0.000   0.983   0.017
B R  4P5TC                124    0.194  44.357   0.460   0.002   0.816   0.182
E N  4P5TC                125    0.576  84.297  -0.571   0.005   0.045   0.951
E S  4P5TC                126    0.678  79.438  -1.312   0.005   0.015   0.979
E K  4P5TC                127    0.563 115.830   0.380   0.003   0.718   0.279
E S  4P5TC                128    0.611  71.633   0.150   0.003   0.718   0.279
B V  4P5TC                129    0.162  24.961   0.808   0.021   0.451   0.528
E A  4P5TC                130    0.487  53.623   0.079   0.021   0.279   0.699
E D  4P5TC                131    0.636  91.662  -0.297   0.019   0.141   0.840
E G  4P5TC                132    0.629  49.534  -1.914   0.020   0.205   0.775
E V  4P5TC                133    0.326  50.183  -0.265   0.021   0.451   0.528
E Y  4P5TC                134    0.507 108.239  -0.598   0.023   0.655   0.322
E E  4P5TC                135    0.406  70.858  -0.743   0.021   0.451   0.528
E T  4P5TC                136    0.330  45.716  -2.089   0.021   0.279   0.699
E S  4P5TC                137    0.504  59.092  -1.214   0.021   0.451   0.528
E F  4P5TC                138    0.400  80.300  -0.365   0.022   0.552   0.426
E F  4P5TC                139    0.306  61.494  -0.179   0.022   0.552   0.426
E V  4P5TC                140    0.412  63.263  -1.028   0.021   0.451   0.528
E N  4P5TC                141    0.563  82.482  -0.719   0.004   0.197   0.799
E R  4P5TC                142    0.675 154.575  -1.596   0.005   0.015   0.979
E D  4P5TC                143    0.686  98.809  -1.328   0.003   0.003   0.994
E Y  4P5TC                144    0.323  68.982  -0.773   0.004   0.138   0.858
B S  4P5TC                145    0.146  17.053   0.288   0.003   0.718   0.279
B F  4P5TC                146    0.058  11.681   0.336   0.001   0.900   0.099
B H  4P5TC                147    0.091  16.535   0.528   0.001   0.959   0.040
B K  4P5TC                148    0.053  10.902   0.092   0.001   0.959   0.040
B L  4P5TC                149    0.102  18.695   0.399   0.001   0.959   0.040
B S  4P5TC                150    0.032   3.704  -0.570   0.001   0.959   0.040
B Y  4P5TC                151    0.113  24.212   0.218   0.001   0.959   0.040
B L  4P5TC                152    0.031   5.658   0.059   0.001   0.959   0.040
E T  4P5TC                153    0.301  41.776   0.544   0.001   0.900   0.099
B F  4P5TC                154    0.065  12.945   0.464   0.003   0.718   0.279
E I  4P5TC                155    0.335  61.901   0.627   0.005   0.262   0.733
E P  4P5TC                156    0.401  56.845  -0.351   0.004   0.085   0.910
E S  4P5TC                157    0.666  78.079  -0.439   0.005   0.015   0.979
E D  4P5TC                158    0.647  93.175  -0.030   0.005   0.015   0.979
E D  4P5TC                159    0.648  93.334  -0.499   0.005   0.015   0.979
E D  4P5TC                160    0.330  47.625   0.106   0.004   0.197   0.799
E I  4P5TC                161    0.429  79.384   1.564   0.003   0.718   0.279
B Y  4P5TC                162    0.053  11.412   0.935   0.001   0.900   0.099
B D  4P5TC                163    0.209  30.146   1.207   0.001   0.959   0.040
B C  4P5TC                164    0.013   1.853   1.425   0.001   0.959   0.040
B K  4P5TC                165    0.185  38.137   1.276   0.001   0.959   0.040
B V  4P5TC                166    0.027   4.165   0.563   0.001   0.959   0.040
E E  4P5TC                167    0.349  60.918   0.737   0.001   0.959   0.040
B H  4P5TC                168    0.086  15.716  -0.294   0.002   0.816   0.182
E W  4P5TC                169    0.482 115.897  -0.384   0.004   0.197   0.799
E G  4P5TC                170    0.508  40.011  -1.089   0.004   0.085   0.910
B L  4P5TC                171    0.242  44.347  -0.297   0.004   0.085   0.910
E E  4P5TC                172    0.670 116.997   0.012   0.005   0.045   0.951
E E  4P5TC                173    0.510  89.167   0.248   0.004   0.085   0.910
E P  4P5TC                174    0.485  68.793  -0.379   0.005   0.262   0.733
B V  4P5TC                175    0.151  23.147   0.608   0.003   0.718   0.279
E L  4P5TC                176    0.366  67.033   0.613   0.001   0.900   0.099
E K  4P5TC                177    0.272  55.909   1.812   0.001   0.959   0.040
E H  4P5TC                178    0.481  87.421   1.134   0.001   0.959   0.040
B W  4P5TC                179    0.098  23.545   0.747   0.002   0.816   0.182
E E  4P5TC                180    0.479  83.611   1.320   0.004   0.616   0.381
E P  4P5TC                181    0.367  52.035  -1.230   0.004   0.197   0.799
E E  4P5TC                182    0.855 149.386   0.510   0.000   0.000   0.999

Explain the output.  Go back.
```

---
